# IgE-Mast cell mediated allergy: a sensor of food quality

**DOI:** 10.1038/s41392-023-01695-y

**Published:** 2023-12-08

**Authors:** Christopher C. Udoye, Rudolf A. Manz

**Affiliations:** Institute for Systemic Inflammation Research, University of Lübeck, Lübeck, Germany

**Keywords:** Medical research, Adaptive immunity

In two recent studies published in *Nature*, Florsheim et al. and Plum et al. reported that allergic-specific reactions induced by immunoglobulin (Ig)E mediated mast cell activation is crucial for the timely induction of antigen-avoidance behaviour.^1,2^ These studies prove the concept that allergic sensitization triggers the formation of avoidance behaviour and define the underlying molecular mechanism.

Food is composed of a great variety of individual components. It often contains noxious substances, typically acting in a dose-dependent manner. Low doses are often tolerated, but ingestion of higher quantities over longer periods might be harmful. Therefore, a variety of physiological sensors exist that act as “food quality control systems”. They use the olfactory, gustatory, and gut chemosensory systems to induce a variety of physiological reactions such as mucus overproduction, increased peristalsis, nausea, vomiting, diarrhoea, or malabsorption, eventually triggering avoidance behaviour.^3^ However, these “classical food quality control systems” can detect only a small number of substances and lack the capacity to adapt to novel challenges.

Type 1 allergic reactions can induce similar symptoms as already known sensors of food quality. This similarity suggested that type 1 allergic reactions may also act as a ‘food quality control system” that allows learning and behaviour adaptation.^3^ The great advantage of involving the immune system would be its ability to recognize a nearly infinite number of distinct substances in a very specific manner, which is an original property of the adaptive immune system. Therefore, in principle, any dietary protein can cause specific allergic sensitization.

Hence, the concept that antibody-mediated allergic responses enable learning and avoidance behaviour was charming but was neither proven nor mechanistically understood. Florsheim et al. and Plum et al. investigated the effects of allergic sensitization on avoidance behaviour in mouse models of food allergy to the model antigen ovalbumin (OVA). Their results show that early type 1 allergic reactions trigger persistent allergen-specific avoidance behaviour, measured by the frequency of licks of OVA containing water. However, mice genetically deficient for IgE or mast cells, and mast cell depleted mice did not develop avoidance behaviour (Fig. 1). These results show that among the many Ig subclasses and cellular responses of the immune reaction to OVA, IgE and mast cells play non-redundant functions in immune mediated food sensing.

**Fig. 1:**
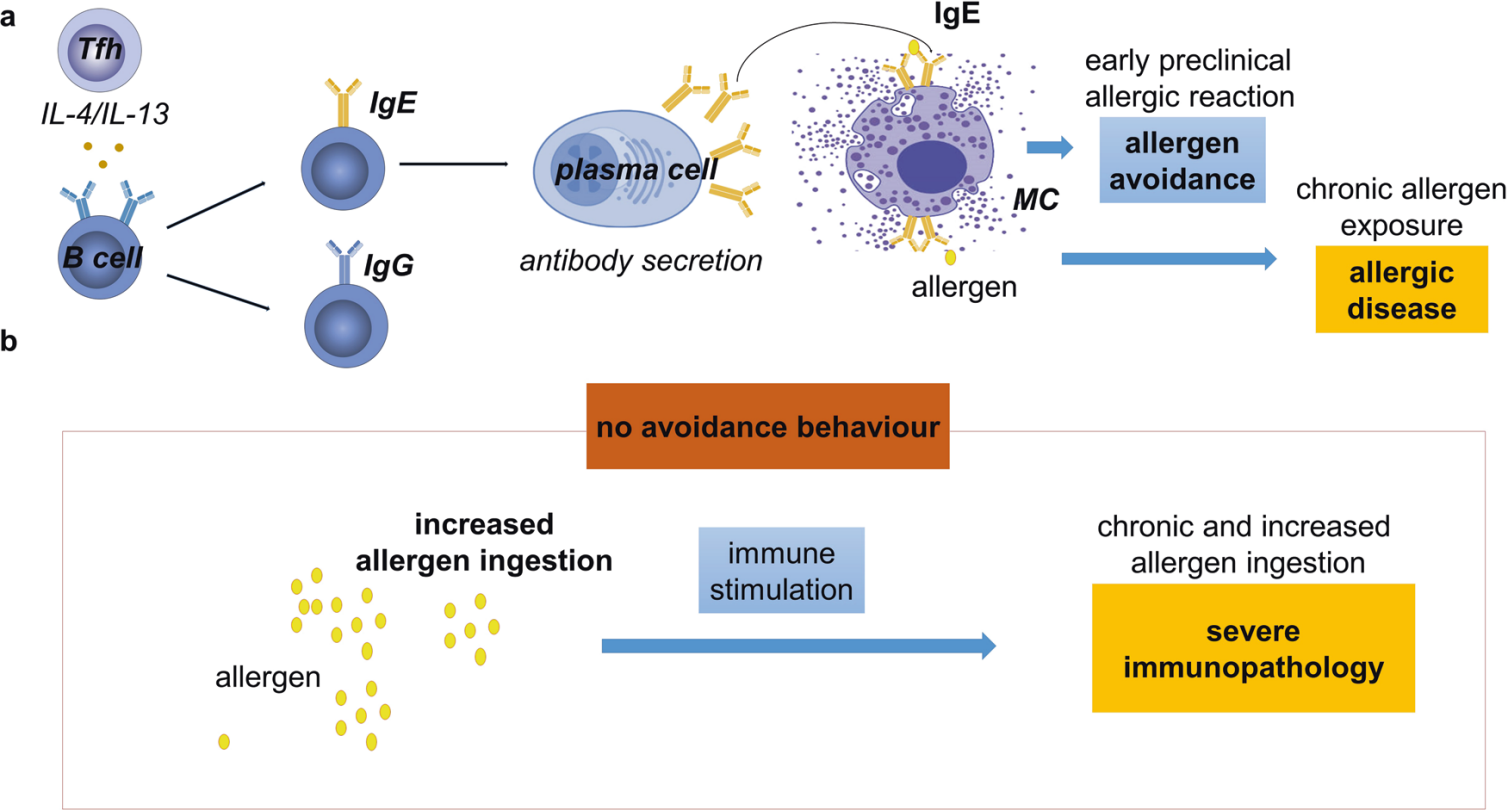
Type 1 allergic reactions promote protective antigen avoidance behaviour via IgE armed mast cells. **a**, In response to allergic stimulation, cytokines from Tfh cells promote antibody class switch and antibody secretion by B cells, which yield antibodies of various isotypes, including IgE and IgG. IgE arms mast cells with an antigen-specific receptor. Already during the early preclinical allergic response, mast cells are sufficiently activated to release leukotrienes, which triggers long-lasting allergen-specific avoidance behaviour. If the allergic stimulation nevertheless persists, IgE may rise and increase its affinity to allergen to trigger strong mast cell activation and allergic disease. **b**, Florsheim et al. showed that In the absence of IgE or mast cells, allergen ingestion is increased, eventually leading to severe immunopathology. Ig Immunoglobulin, IL Interleukin, MC mast cell, Tfh T follicular helper

IgE and mast cells belong to the same effector cascade of type 1 allergic reactions. IgE binds to the FcεRI on mast cells, arming these cells with an antigen/allergen specific receptor. IgE production is dependent on IL-4 from T follicular helper (Tfh) cells. This cytokine is sufficient to induce low affinity IgE, which however does not induce severe allergic symptoms, while additional cytokines from Tfh13 cells, including IL-13 and IL-21, are required for the development of high affinity IgE and anaphylaxis, a severe and potentially lethal condition.^4^ High affinity IgE-mediated mast cell activation is the major mechanism for the induction of severe type-1 allergic reactions. But murine IgG1 (human IgG4) antibodies which are also induced by IL-4, can trigger anaphylaxis too, though only in the presence of higher amounts of antigen. Antibodies of the subclasses IgG2 and IgG3 are not IL-4 dependent, may not contribute to the pathology of type 1 allergic reactions, but can mediate severe inflammation via the activation of the complement system and various innate effector cells. The studies by Florsheim et al. and Plum and colleagues now show that mice lacking IgE do not avoid allergen ingestion. While most allergic symptoms are absent in IgE deficient mice, even after forced uptake of high amounts of allergen, they still develop severe anaphylaxis, probably involving antibodies of other subclasses. Together, these data are consistent with the notion that IgE is critical for the development of allergic symptoms under physiologic conditions, when allergen avoidance behavior prevents the uptake of the high allergen doses required to induce non-IgE-mediated anaphylaxis. This work reveals a protective role of IgE-mediated mast cell activation, acting via modification of behaviour.

How exactly mast cell activation translates into the modification of behaviours is not yet completely understood. Florsheim et al. showed that even early allergic symptoms are associated with the activation of areas of the brain involved in the response to aversive stimuli. Importantly, the results indicated that the induction of allergen avoidance behaviour required only mild allergic reactions mediated by IgE, which precede the development of gut allergic inflammation. Evidence was provided, that activated mast cells affect behaviour through the release of cysteinyl leukotrienes and the induction of growth and differentiation factor 15 by colonic epithelial cells, eventually sensed by the nervous system. But the target cells within the nervous system remain to be elucidated.

Florsheim et al. suggest that the early IgE-mediated allergic reaction triggers avoidance behaviour while chronic allergen ingestion results in IgE-mediated disease.^1^

In accordance with, but also extending this view, we believe that the balance between distinct immune reactions within one individual is the key to understand the role of IgE armed mast cells in immunoprotection and allergy development. The ratios between the levels of allergen specific IgE and the levels of allergen specific antibodies of other subclasses, correlate better with the development of severe allergic symptoms than the levels of IgE alone.^5^ This reflects the fact that antibodies of other subclasses such as IgG1 or IgA can inhibit the severe allergic reactions induced by high-affinity IgE.

The generation of high levels of IgA and IgG and the production of low affinity IgE precedes the formation of pathogenic high affinity IgE. Hence, under conditions when low affinity IgE and antibodies of non-IgE subclasses prevail, it may primarily act as a food quality control system. This is the case at the early, preclinical stage of the allergic reaction. In addition, this mechanism may also be relevant in non-allergic individuals, who nevertheless produce subclinical quantities of IgE, potentially sufficient to cause mild mast cell activation and behavioural change, but without triggering allergic pathology.

## References

[1] Florsheim EB (2023). Immune sensing of food allergens promotes avoidance behaviour. Nature.

[2] Plum T (2023). Mast cells link immune sensing to antigen-avoidance behaviour. Nature.

[3] Florsheim EB, Sullivan ZA, Khoury-Hanold W, Medzhitov R (2021). Food allergy as a biological food quality control system. Cell.

[4] Gowthaman U (2019). Identification of a T follicular helper cell subset that drives anaphylactic IgE. Science.

[5] Udoye CC (2022). B-cell receptor physical properties affect relative IgG1 and IgE responses in mouse egg allergy. Mucosal Immunol..

